# Insight into the Antioxidant Activity of 1,8-Dihydroxynaphthalene Allomelanin Nanoparticles

**DOI:** 10.3390/antiox12081511

**Published:** 2023-07-28

**Authors:** Alexandra Mavridi-Printezi, Fabio Mollica, Rosa Lucernati, Marco Montalti, Riccardo Amorati

**Affiliations:** Department of Chemistry “Giacomo Ciamician”, University of Bologna, Via Selmi 2, 40126 Bologna, Italy; alexandra.mavridi2@unibo.it (A.M.-P.); fabio.mollica3@unibo.it (F.M.);

**Keywords:** antioxidants, nanoparticles, melanin, allomelanin, polydopamine, ROS, oxidative stress, peroxidation, radicals, superoxide, hydroperoxyl radical

## Abstract

Melanins are stable and non-toxic pigments with great potential as chemopreventive agents against oxidative stress for medical and cosmetic applications. Allomelanin is a class of nitrogen-free melanin often found in fungi. The artificial allomelanin obtained by the polymerization of 1,8-dihydroxynaphthalene (DHN), poly-DHN (PDHN), has been recently indicated as a better radical quencher than polydopamine (PDA), a melanin model obtained by the polymerization of dopamine (DA); however, the chemical mechanisms underlying this difference are unclear. Here we investigate, by experimental and theoretical methods, the ability of PDHN nanoparticles (PDHN-NP), in comparison to PDA-NP, to trap alkylperoxyl (ROO^•^) and hydroperoxyl (HOO^•^) radicals that are involved in the propagation of peroxidation in real conditions. Our results demonstrate that PDHN-NP present a higher antioxidant efficiency with respect to PDA-NP against ROO^•^ in water at pH 7.4 and against mixed ROO^•^ and HOO^•^ in acetonitrile, showing catalytic cross-termination activity. The antioxidant capacity of PDHN-NP in water is 0.8 mmol/g (ROO^•^ radicals quenched by 1 g of PDHN-NP), with a rate constant of 3 × 10^5^ M^−1^ s^−1^ for each reactive moiety. Quantum-mechanical calculations revealed that, thanks to the formation of a H-bond network, the quinones in PDHN-NP have a high affinity for H-atoms, thus justifying the high reactivity of PDHN-NP with HOO^•^ observed experimentally.

## 1. Introduction

**Figure 1:**
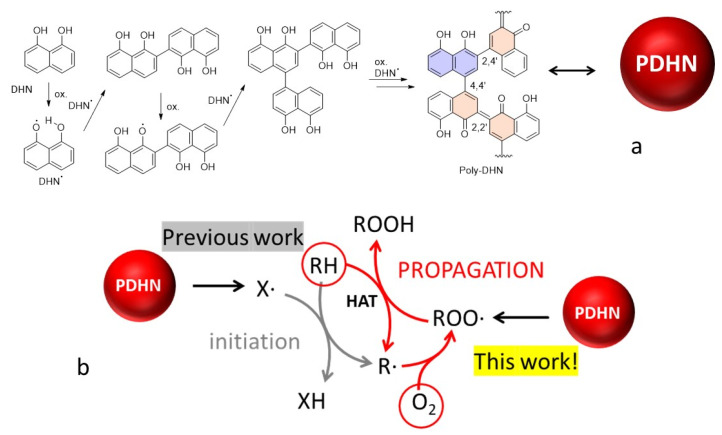
(**a**) Oxidative polymerization of DHN with formation of PDHN-NP, with a focus on the presence of reduced units inside the polymer; (**b**) schematic process of autoxidation of biomolecules (RH) by atmospheric O_2_ mediated by radicals, and possible inhibition effects by PDHN-NP.

Reactive oxygen species (ROS) play an important role in Nature as physiological signaling agents [1,2]. However, human life span, aging, and several diseases have been recognized to be closely related to the results of oxidative processes [3,4,5]. Due to this, ROS regulation is a fundamental issue for human health preservation, while redox regulation has become a powerful tool for preventive medicine and the treatment of important pathologies. In this framework, governing the levels of ROS and reactive nitrogen species (RNS), normally involved in physiological signaling, is fundamental to avoiding oxidative stress [6]. The species able to prevent damages caused by ROS and RNS, generally known as antioxidants (AOX), have been proposed in the last few decades as potential therapeutic agents [7,8,9]. Beside this, a very dramatic negative effect of the interaction of radicals with living tissues is, as in the case of ferroptosis [10], to activate the oxidation of biomolecules by atmospheric oxygen, a process itself thermodynamically favored and only accelerated by the catalytic effect of radicals [11]. The process of initiation, schematized in Figure 1, is in fact followed by a chain reaction, the propagation, that involves the alkylperoxyl radical (ROO^•^), a radical that normally differs from the initiator X^•^ [12,13,14].

Due to the fact that the ROS family is constituted by different species with various physiological roles, an unspecific AOX activity is not suitable to guarantee a beneficial therapeutic action. In general, the same AOX is not equally effective in contrasting any kind of radical or highly reactive species [15,16]. For this reason, understanding the mechanism at the base of the activity of new AOX is essential [17,18,19]. With respect to traditional “small-molecule” AOX, nanomaterials offer specific features and advantages [20,21,22,23,24,25]. To date, there are examples of “nano-antioxidants” displaying a reduced migration inside the protected material, a better resistance to degradation due to heat and O_2_, a better bioavailability, the possibility of being controlled through magnetic fields, and the ability to reach specific organs via controlled disassembly [26,27,28,29,30,31].

Melanins are a broad family of natural and synthetic materials with interesting properties such as surface covering, adhesion enhancement, redox activity, and metal chelation, to mention only a few [32,33]. Melanins derive from the oxidative polymerization of phenols bearing amino substituents (such as tyrosine or dopamine), while allomelanins, naturally found in certain fungi, are characterized by the absence of nitrogen in the precursor [34,35,36,37]. Melanin-like materials can also be obtained by the copolymerization of phenols with bifunctional amines [38]. Especially due to their high biocompatibility and because of the presence of reduced and oxidized phenol moieties, melanin-like nanomaterials are promising AOX agents and show defense against a wide range of ROS [39,40,41]. However, most of the investigations have been devoted to eumelanin, which is the melanin naturally present in humans and deriving from enzymatically catalyzed oxidation of tyrosine, and to its synthetic analogue, polydopamine (PDA), which can be obtained by oxidative polymerization of dopamine in water [42], while little is known about other kinds of melanins and their artificial mimics. Artificial allomelanin-mimicking nanoparticles (NP), obtained by polymerization of 1,8-dihydroxynaphthalene (DHN) and therefore named poly-1,8-dihydroxynaphthalene nanoparticles (PDHN-NP) (Figure 1), have been reported to be better scavengers of 2,2-diphenyl-1-picrylhydrazyl (DPPH) radicals than PDA-NP [43,44,45,46]. Additionally, PDHN-NP can prevent the radical damage generated by UV irradiation of keratinocytes, presumably by avoiding the formation of initiating radicals X^•^ [44]. Unfortunately, DPPH is a model radical with a little resemblance to the biologically relevant ROO^•^ radicals [47], thus it is still not known if PDHN-NP can prevent the propagation of the oxidation process shown in Figure 1 or what is the mechanism of its antioxidant activity.

Recently, some of us have found that PDA-NP can slow down the peroxidation of organic materials only in cases in which the chain propagation involves both ROO^•^ and HOO^•^ radicals [12]. This condition is indeed common to many real systems, including the autoxidation of molecules with alcohol and amine functional groups that can be found, for example, on the surface of proteins. HOO^•^ can regenerate phenolic antioxidants by prolonging their activity because, unlike ROO^•^, HOO^•^ has a double-faced oxidizing and reducing character [48]. These results have shed a new light on the mechanism of antioxidant activity of PDA and demonstrated the importance of focusing the attention on those radicals that are formed during the autoxidation of real substrates. In this work, we aim at filling the information gap regarding the reactivity of PDHN-NP with ROO^•^ and HOO^•^ radicals, as this material has been previously investigated only by DPPH quenching experiments. We demonstrate, for the first time, when and how PDHN-NP can terminate the propagation of the oxidation process schematized in Figure 1 providing an insight into the AOX mechanism of action of fungus melanin. Moreover, the AOX properties of the allomelanin model are compared with those of artificial eumelanin using ROS found in “realistic” oxidative stress conditions.

## 2. Materials and Methods

### 2.1. Materials

All reagents, solvents, and chemicals were purchased from Sigma-Aldrich (Steinheim, Germany) and used without modification, unless otherwise stated. Gamma-terpinene was percolated onto alumina and silica before each experiment. 2,2′-azobis(2-methylpropionitrile) (AIBN), was crystallized from methanol. Tetrahydrofuran (THF) was purified by distillation, and the water was Millipore grade (resistivity < 18 MΩ).

### 2.2. UV-Vis Absorption Spectroscopy

The experiments were carried out in air-equilibrated solutions at 25 °C. UV-Vis absorbance spectra were recorded with a Perkin-Elmer Lambda 650 (Boston, MA, USA) spectrophotometer using quartz cells (Hellma, Müllheim, Germany) with a path length of 1.0 cm or UV disposable cuvettes purchased by BRAND (Wertheim, Germany).

### 2.3. Dynamic Light Scattering (DLS)

DLS measurements were performed with Zetasizer Nano ZS Malvern Panalytical (Worcestershire Malvern, United Kingdom) using PMMA semi-micro cuvettes (BRAND Wertheim, Germany) and a folded capillary Zeta cell (Malvern, Worcestershire Malvern, United Kingdom).

### 2.4. Transmission Electron Microscopy (TEM)

TEM images were received using a Philips TEM CM 100 electron microscope (Eindhoven, Holland) at an accelerating voltage of 80 kV. For the acquisition, the samples were deposited on Formvar on 400 mesh Cu grids supplied by TED PELLA INC. (Redding, CA, USA).

### 2.5. Electron Paramagnetic Resonance (EPR)

The EPR spectra were collected at 25 °C with a MiniScope MS 5000 (Magnettech, Bruker, Billerica, MA, USA) in glass capillary tubes. The radical concentration was determined by comparing the double integral of its EPR spectrum to that of a reference DPPH solution. Spectral simulation was performed using EasySpin software with the graphical interface SimLabel. as previously reported [13].

### 2.6. AOX Properties

The kinetics of the reaction with alkylperoxyl radicals were studied by autoxidation experiments in differential oxygen-uptake apparatus based on a Validyne DP 15 pressure transducer built in our laboratory [49]. O_2_-consumption experiments were performed in duplicate. The values of the rate constants and of the stoichiometries of radical trapping are expressed as the average ± SD (standard deviation).

#### 2.6.1. Autoxidation of Tetrahydrofuran (THF) in Water at pH 7.4 [50]

The samples consisted of the hydrosoluble 4,4′-azobis(4-cyanovaleric acid) sodium salt (ABCV) (53 mM), THF (10% *v*/*v*, 1.2 M), and 0.1 M phosphate buffer (pH 7.4) at a temperature of 30 °C under vigorous stirring. By using the α-tocopherol hydrosoluble analogue Trolox as a reference antioxidant (having *n* = 2), the rate of radical initiation was calculated, using the relation *R*_i_ = *n*[Antiox.]/τ, where τ is the duration of the inhibition period, as *R*_i_ = 5.2 × 10^−9^ Ms^−1^ [51].

#### 2.6.2. Autoxidation of Styrene and γ-Terpinene in Acetonitrile [50]

The samples consisted of styrene (2 mL, 4.3 M), azobisisobutyronitrile (AIBN) (25 mM), variable amounts of γ-terpinene and the investigated antioxidant at a temperature of 30 °C under vigorous stirring. The structural analogue of α-tocopherol, 2,2,5,7,8-pentamethyl-6-chromanol (PMHC), was used as a reference antioxidant. The rate of initiation was measured as *R*_i_ = 3.2 × 10^−9^ Ms^−1^.

### 2.7. Synthesis of PDHN

PDHN-NP was prepared by oxidative polymerization of 1,8-DHN in solution in the presence of potassium permanganate (KMnO_4_) at room temperature [44]. More in detail, 20 mg of 1,8-DHN were dissolved in 1.27 mL of acetonitrile (ACN) by sonication in a 50 mL round bottom flask. Furthermore, 12.06 mL of HOAc-NaOAc buffer 0.1 M (pH = 3.8) were added to the round bottom flask together with 469 μL of 0.2 M KMnO_4_ under vigorous stirring. After 24 h, the nanoparticles were retrieved by centrifugation at 11.000 rpm for 10 min and were washed five times with ultrapure water using the same centrifugation speed. After the purification, the nanoparticles were redispersed either in water or ACN.

### 2.8. Theoretical Calculations

The geometries were optimized at the PBE1PBE/6-31+g(d,p) [52] level by using Gaussian 09 [53]. The absence of imaginary frequencies was checked by a frequency calculation at the same level. The electronic energy was calculated by a single-point calculation at the PBE1PBE/6-311++g(2d,2p) level [52]. The H-atom affinity of the quinones was determined as the negative value of the Bond Dissociation Enthalpy of the semiquinone, BDE(QH^•^). The BDE values were determined by using the isodesmic approach shown in Equations (1) and (2) [54], which consists of calculating the BDE difference (BDE_calc_) between the investigated molecule and a reference whose BDE has been experimentally determined (BDE_exp_).

BDE(QH^•^) = BDE_exp_(PhOH) + ΔBDE_cal_ (1)

ΔBDE_calc_ = [ΔH(QH^•^) − ΔH(Q)] − [ΔH(PhOH) − ΔH(PhO^•^)] (2)

In Equations (1) and (2), ΔH is the sum of the single-point electronic energy and thermal enthalpy from frequency calculations in the gas phase, and BDE_exp_(PhOH) is the experimental BDE(OH) of phenol in benzene (87.6 kcal/mol) [55,56]. The optimized geometries are available in the Supplementary Material.

## 3. Results and Discussion

### 3.1. Synthesis and Characterization

**Figure 2:**
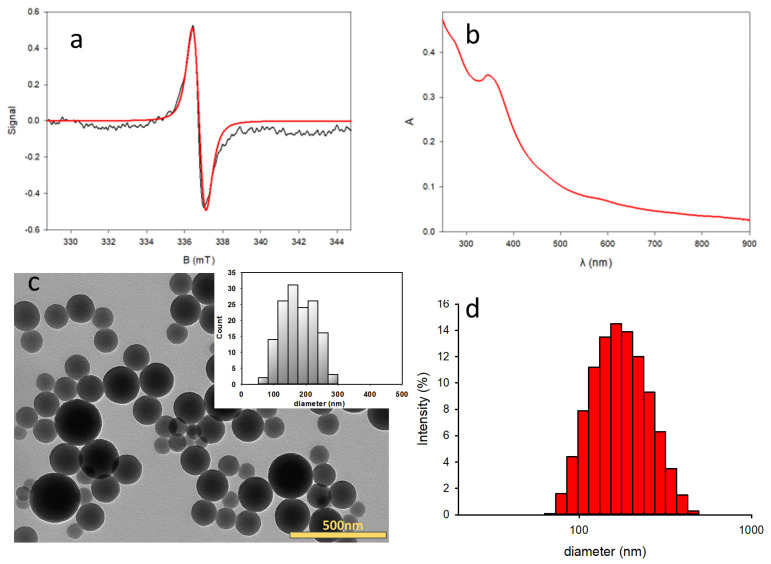
(**a**) EPR spectrum (X-band) of PDHN-NP in water. Black line: experimental spectrum, red line: simulated by EasySpin software; (**b**) UV-Vis absorbance spectrum of PDHN-NP in water. (**c**) TEM images of PDHN-NP. The size distribution of the NPs given by TEM is plotted as an inset; (**d**) PDHN-NP size distribution measured by DLS.

PDHN-NP were prepared by oxidative polymerization of 1,8-dihydroxynaphthalene (DHN) in solution in the presence of potassium permanganate at room temperature (see Section 2.6) [44]. Their synthesis involves the oxidation of the DHN precursor to give the intermediate species shown in Figure 2, eventually leading to the formation of spherical NP. The UV-Vis absorbance spectra of the PDHN-NP in water (Figure 2b) show a broad band absorption with a peak at around 350 nm, similar to what is reported in the literature [46,57]. The size of PDHN-NP was further verified by TEM, indicating a size distribution between 150 and 280 nm (Figure 2c). The hydrodynamic diameter of the NP, measured by DLS, was 230 nm and the polydispersity index was 0.1 (Figure 2d). EPR spectroscopy revealed the presence, within PDHN-NP, of persistent radicals, which are commonly detected in melanins as the result of the comproportionation of quinones and phenols coexisting in the polymer [43]. The EPR spectrum consists of a single broad line with a *g* factor of 2.00372 (similar to that of other melanins) and corresponding to a spin density of 1.9 ± 0.5 μmol g^−1^ (see Figure 2a).

### 3.2. Kinetic Studies of the Reaction with ROO^•^ and HOO^•^

**Figure 3:**
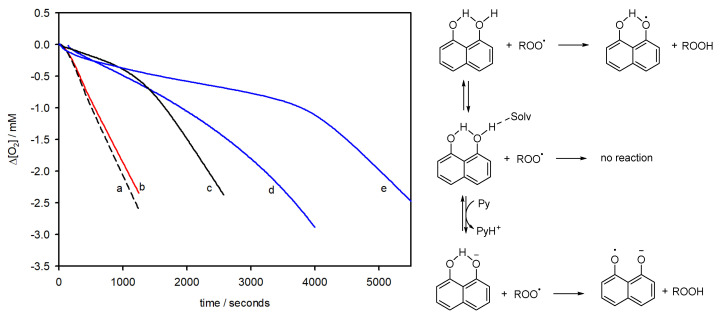
Representative O_2_ consumption measured during the autoxidation of styrene (4.3 M) in MeCN, initiated by AIBN (25 mM) at 30 °C. (**a**) Without antioxidants, (**b**) with PDHN-NP 16 μg/mL; (**c**) PMHC 2.5 μM; (**d**) DHN 5.0 μM; (**e**) DHN 5.0 μM with pyridine 62 mM. Left: mechanism of antioxidant activity of DHN.

In order to investigate the different reactivity with ROO^•^, the reaction of PDHN-NP was first studied against the autoxidation of a model organic substrate (styrene) initiated by the thermal decomposition of AIBN (2,2′-azobis(2-methylpropionitrile)) at 30 °C in acetonitrile (see Figure 3) [12,13]. This reactive system is often used in the kinetic investigations of AOX because it reproduces an oxidative stress condition where a specific radical, ROO^•^ is generated continuously and the reaction with the AOX (*k*_inh_) has to compete with chain propagation (*k*_p_) [14]. Additionally, the chain inhibition can be conveniently detected by following the consumption of O_2_. Acetonitrile (MeCN) is a polar aprotic solvent that can solubilize both lipophilic oxidizable substrates and polar antioxidants, and it has been successfully used to disperse phenolic polymers and other nanoantioxidants [12]. Hence, in the absence of AOX, the O_2_ consumption is linear, while after the addition of an AOX able to quench ROO^•^ (AH), an inhibited period is observed. From the slope of O_2_ consumption and the duration of the inhibited period, the rate constant for the reaction with ROO^•^ (*k*_inh_) and the number of ROO^•^ trapped by each AH (*n*) can be obtained by Equations (3) and (4), respectively, where *τ* is the duration of the inhibited period and *R*_i_ is the rate of ROO^•^ production by the initiator [14].

(3) $$-d[O_{2}]/dt=(k_{p}\left[RH\right]R_{i})/(nk_{inh}\left[AH\right])$$

(4) $$R_{i}=n\left[AH\right]/\tau$$

**Figure 4:**
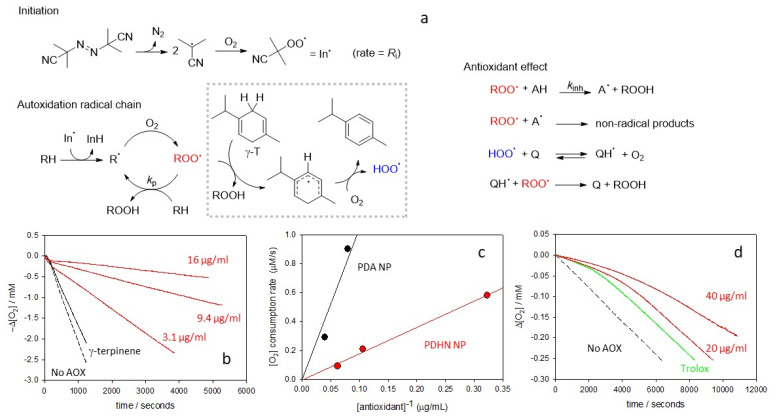
(**a**) General reaction scheme for the autoxidation of organic substrates (RH) propagated by ROO^•^ and, in the presence of γ-terpinene (γ-T), HOO^•^ radicals; and inhibition by H-atom donating AOX (AH) or quinones (Q). (**b**) Representative O_2_ consumption measured during the autoxidation of styrene (4.3 M) in acetonitrile, initiated by AIBN (25 mM) at 30 °C without AOX, with γ-T (31 mM), and different amounts of PDHN-NP: 3.1 μg/mL, 9.4 μg/mL and 16 μg/mL, all with γ-T (31 mM). (**c**) Comparison between the radical trapping activity of PDHN-NP (slope = 1.8 × 10^−6^ M^2^ s^−1^) and PDA-NP (data from ref. [12], slope = 1.1 × 10^−5^ M^2^ s^−1^), (**d**) O_2_ consumption measured during the autoxidation of THF (1.2 M) in phosphate buffer pH 7.4, initiated by ABCV (53 mM) at 30 °C: without AOX, with Trolox 10 μM, with PDHN-NP 20 μg/mL and with PDHN-NP 40 μg/mL.

The results of these experiments (see Figure 3) show that PDHN-NP does not act as an AOX in MeCN (line b), while its precursor DHN does (line d). This result is similar to that obtained for PDA-NP, which is also unreactive toward ROO^•^ in MeCN [12]. The *k*_inh_ of DHN in MeCN is calculated as (1.0 ± 0.2) × 10^5^ M^−1^ s^−1^ and its *n* value is 2.0 ± 0.2. The *k*_inh_ value is lower than that previously measured in chlorobenzene (4.3 × 10^6^ M^−1^ s^−1^) [58] and is due to the well-known kinetic solvent effect of H-bond accepting solvents on H-atom transfer (HAT) reactions from polar X-H groups (X = O, N), thus demonstrating that the reaction between ROO^•^ and DHN follows a HAT mechanism (see scheme in Figure 3) [59]. Interestingly, the addition of pyridine, a weak base, leads to an increased *k*_inh_ value of (1.7 ± 0.2) × 10^5^ M^−1^ s^−1^, reasonably because it forms small amounts of the DHN anion with an augmented reactivity toward ROO^•^. A possible mechanism explaining this result is reported in the scheme in Figure 4, which is based on the previously observed antioxidant activity increase in ascorbic acid (vitamin C) in the presence of bases [60,61].

To explore other mechanisms that could explain the antioxidant activity of PDHN NP, we studied the autoxidation of styrene in MeCN in the presence of a little amount of the pro-aromatic derivative γ-terpinene, which is a natural highly oxidizable hydrocarbon contained in lime and oregano essential oils, which affords the aromatic hydrocarbon *p*-cymene with the concomitant production of HOO^•^ radicals that propagate the oxidative chain (see Figure 4) [61,62]. This experimental setup reproduces a feature common to many real systems, namely the simultaneous presence of mixed (oxidizing) ROO^•^ and (reducing) HOO^•^ radicals [48]. For example, at the membrane interface, HOO^•^ can regenerate antioxidants, prolonging their activity against ROO^•^ [63].

When using PDHN-NP as an AOX in the styrene and γ-terpinene mixture (Figure 4), a good antioxidant activity proportional to PDHN-NP concentration is observed. When plotting the slope of O_2_ consumption against the inverse of the concentration of the studied material (see Equation (3) and Figure 4b), a straight line is obtained, whose slope is inversely proportional to the AOX activity. This analysis showed that the ROO^•^/HOO^•^ quenching by artificial allomelanin-like NP is much greater than that observed previously with PDA eumelanin-like NP [12]. We explain this antioxidant effect by the mechanism reported in Figure 4a [64]. Briefly, the HOO^•^ radicals react with the quinones present in PDHN-NP, generating transient semiquinone radicals that, in turn, are able to trap further HOO^•^ or ROO^•^ radicals. The absence of a clearly defined inhibition period in the experiments reported in Figure 4b suggests that PDHN-NP has a catalytic effect.

Finally, we investigated the AOX activity in an aqueous environment, which is relevant to the oxidative damage of hydrophilic macromolecules. The AOX activity of PDHN-NP was investigated in water at pH 7.4 by using tetrahydrofuran (THF) as an oxidizable substrate and 4,4′-azobis(4-cyanovaleric acid) (ABCV) as an initiator. The water-soluble analogue of α-tocopherol, Trolox, was used as a reference (*k*_inh_ = 4.1 × 10^5^ M^−1^ s^−1^, *n* = 2) [50,65]. In these experiments, PDHN-NP behaved as a moderately good AOX, being able, at a concentration of 20–40 μg/mL, to provide an inhibition similar to that of Trolox (Figure 4). Under identical conditions, PDA-NP showed negligible AOX activity [13]. From the duration of the PDHN-NP inhibition period, it is possible to calculate the ROO^•^ scavenging ability as 0.8 mmol of ROO^•^ radicals quenched by 1 g of PDHN-NP. This value, which is about 20 times smaller than that obtained previously by the 2,2-diphenyl-1-picrylhydrazyl (DPPH^•^) test [43,46], represents the concentration of the radical-quenching moieties that can react fast enough with ROO^•^ radicals before chain-propagation occurs (ROO^•^ + THF → ROOH + THF_-H•_) [66]. The *k*_inh_ value of the active moieties of PDHN-NP can be estimated as 3 × 10^5^ M^−1^ s^−1^.

The structure of PDHN-NP reported in Figure 1a [52,67] provides some clues about the nature of such reduced moieties. While polymerization by phenoxyl radical coupling should provide a completely reduced chain [52], nevertheless, the oxidative conditions of the synthesis are expected to also affect the polymer chain. As oxidation consists of the formation of an extended quinone between nearby DHN units, it is possible that some reduced DHN units remain trapped among couples of oxidized DHN units, as shown by the purple structure in Figure 1a. On the other hand, the phenolic moieties present in an oxidized DHN unit are not expected to contribute to the antioxidant effect because of the exceedingly negative effect of an *ortho* carbonyl group on the H-atom donation of phenols [68].

The monomer DHN, instead, behaves as a better antioxidant than Trolox, with a *k*_inh_ in water of 1.1 × 10^6^ M ^−1^ s ^−1^ and *n* of 3.5. This result is not surprising because DHN is almost completely dissociated at pH 7.4 (p*K*_a_ = 6.2) [69] and deprotonated phenols are known to have a high rate constant of reaction with ROO^•^ [50]. The relatively high stoichiometric coefficient observed in water can be explained by the tendency of the phenoxyl radicals of DHN to dimerize with the formation of quinones, which may undergo nucleophilic attack by the solvent to regenerate active phenolic groups.

### 3.3. Mechanism of the Reaction with HOO^•^

**Figure 5:**
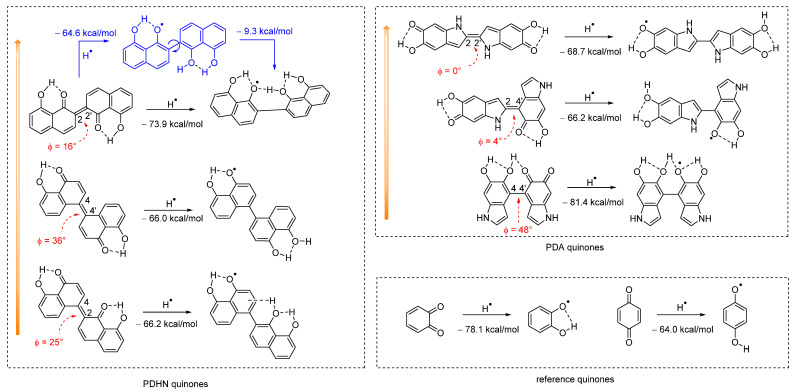
Calculated H-atom affinity of the quinones present in PDHN-NP and PDA-NP. The orange arrows indicate the likelihood of finding the dimers in the polymer. The ϕ angles (in red) represent the dihedral angles formed between two adjacent DHN or DA units.

To understand at a molecular level the different behavior of PDHN-NP and PDA-NP, we investigated by theoretical calculations the ability of the quinones, putatively present in these materials, to accept the H-atom donated by the HOO^•^ (see Figure 5). We have shown previously that 1,2-benzoquinones are better AOX than 1,4-benzoquinones in the presence of HOO^•^ because the former have a higher affinity for H-atoms [12].

The structures used in the calculations were obtained by considering that, during the formation of the polymer, the coupling of DHN radicals can occur principally at the 2 and 4 positions, giving rise to 2,2′, 2,4′, and 4,4′ dimers (see Figure 1), which upon oxidation provide the corresponding quinones [52,67,70]. To compare PDHN-NP to PDA-NP, we also considered the quinones arising from the oxidation of the dimers of 5,6-dihydroxyindole (DHI), which is the main intermediate of dopamine (DA) oxidation. The DHI dimers are linked preferentially at the 2,2′, 2,4′, and 4,4′ positions, following the spin distribution in the DHI radical (for numbering, see Figure 5) [71,72]. However, because of steric requirements, some dimers are expected to be formed more easily than others. Although an experimental quantification is not available, theoretical studies recently appearing in the literature have shown that the stability order of DHI and DHN dimers is (coincidentally) 2,2′ > 2,4′ > 4,4′ [70,71,72]. In Figure 5, the H-atom affinity of the various quinones is reported in order of the likelihood of their presence in the materials. The 1,2 and 1,4 benzoquinones have also been considered in order to have a reference of high and low H-atom affinity, respectively, leading to very different antioxidant effects as previously discussed.

Both in PDHN-NP and PDA-NP, quinones originating from the 2,2′ and 4,4′ dimers have a high affinity for H^•^, with values of −73.9 and −81.4 kcal/mol, respectively. However, while in the case of PDHN-NP the 2,2′ dimer is the most abundant one, in the case of PDA the 4,4′ is the less abundant one. All the other quinones have a similar low affinity for H^•^, ranging between 66 and 69 kcal/mol. Interestingly, the preferred conformation of the quinone of the DHN 2,2′ dimer is “*trans*”, while that of the corresponding semiquinone is “*cis*”. Calculations show that, after the reaction with HOO^•^, the semiquinone undergoes a geometric rearrangement (shown in blue in Figure 5) that allows tightly binding the H-atom, thus increasing the H-atom affinity by about 9 kcal/mol. These results indicate that the higher HOO^•^ trapping ability of PDHN-NP derives from the relatively high H-atom affinity of its most representative quinone, originating from the 2,2′-DHN dimer.

Furthermore, theoretical calculations shed some interesting light on the PDHN and PDA polymers’ structures. The dihedral angle between the two adjacent DHN or DA units in the oxidized dimers is represented by ϕ in Figure 5. The ϕ angle of the quinones in the case of PDA is almost zero, being 0° and 4° for the 2-2′ and for the 2-4′ dimers, respectively, because of the low steric crowding due to the five-membered ring. In contrast, in the case of PDHN, the ϕ angle of the quinones is large, ranging from 16° for the most stable 2-2′ dimer to 36° for the 2-4′ dimer. The strong steric repulsion between the six-membered rings is the cause of this augmented dihedral angle respecting PDA. Based on these findings, it is possible to hypothesize that the nonplanar arrangement of the quinones in PDHN causes a polymer’s disordered structure, which increases the porosity and permits the diffusion of free radicals. On the other hand, the formation of flat quinones with the 2-2′ and 2-4′ links in the case of PDA results in the formation of densely packed aggregates with a low reactivity toward HOO^•^. The 4,4′ DHI dimer, which would have the highest HOO^•^ trapping activity and a large ϕ angle, is only a minor component of PDA, thus explaining the smaller AOX activity of PDA-NP than PDHN-NP.

## 4. Conclusions

In conclusion, in this work, we have contributed to the clarification of the antioxidant activity of PDHN-NP, which represents the most studied allomelanin model. In this study, we investigated the reactivity toward ROO^•^ and HOO^•^ radicals, which are significant radicals for the autoxidation of organic materials. This differs from earlier research, which measured the antioxidant activity only using the decay of the stable radical DPPH. In water, PDHN-NP are able to block the oxidative chain process thanks to the presence of residual phenolic groups, whose rate constant of reaction with ROO^•^ is 3 × 10^5^ M^−1^ s^−1^ (referred to as each reactive moiety). This outcome contrasts sharply with what was previously discovered in the case of PDA-NP, which had no antioxidant effect in water [13]. This is likely due to the PDA polymer’s tighter packing or a lower level of trapped phenolic units. Given the oxidative conditions used for the synthesis, PDHN-NP are not surprisingly less antioxidant than the DHN monomer.

An investigation of acetonitrile gave important insight about the antioxidant mechanism. While PDHN-NP do not directly react with ROO^•^, in the presence of hydroperoxyl radicals (HOO^•^, i.e., protonated superoxide), PDHN-NP are able to catalyze the cross dismutation of HOO^•^ and ROO^•^ radicals, leading to a prolonged inhibition of the autoxidation. In this regard, PDHN-NP were more active than PDA-NP. Theoretical calculations clarified the origin of this difference, suggesting that the extended quinones in PDHN have a higher H-atom affinity than PDA ones and therefore a faster reaction with HOO^•^ radicals. The quinone of the 2-2′ dimer in PDHN can stabilize the H-atom by a network of H-bonds and by a geometrical rearrangement that increases the H^•^ affinity by 9.3 kcal/mol.

These findings suggest that PDHN-NP may be useful to protect substrates whose autoxidation is also mediated by HOO^•^ radicals, such as amines, alcohols, and 1,2 or 1,4 cyclohexadiene derivatives [48]. As HOO^•^ and its conjugated base superoxide (O_2_^•−^) are commonly present in biological systems as a result of O_2_^•−^ leakage from the respiratory chain and as an effect of NADPH oxidase (NOX) activity [48], it can be envisaged that PDHN-NP may be capable of capturing reducing equivalents from superoxide and of using them to stop the peroxidation of important biomolecules in a catalytic fashion [73]. This antioxidant activity may act in synergy with the known protective effect against UV light. Although further work is needed to fully clarify all these exciting aspects, these results pose the rational basis for the potential utility of artificial allomelanin-like PDHN-NP as an antioxidant in protective bio-coatings and in sunscreens.

## Data Availability

Data is contained within the article and Supplementary Material.

## Supplementary Materials

The following supporting information can be downloaded at: https://www.mdpi.com/article/10.3390/antiox12081511/s1, optimized geometries from theoretical calculation.
