# Supplementary material for: Insight into the Antioxidant Activity of 1,8-Dihydroxynaphthalene Allomelanin Nanoparticles
Source: Antioxidants (Basel). 2023 Jul 28;12(8):1511. doi: 10.3390/antiox12081511 (PMC10451768; doi:10.3390/antiox12081511)
Supplement: Supplementary file 1 [file antioxidants-12-01511-s001.zip › antioxidants-2517912-supplementary.pdf]

Supplementary Materials

Insight into the Antioxidant Activity of 1,8-Dihydroxynaphthalene Allomelanin Nanoparticles

*Alexandra Mavridi-Printezi, Fabio Mollica, Rosa Lucernati, Marco Montalti * and Riccardo Amorati **

S.1 Cartesian coordinates

PBE1PBE/6-31+g(d,p) int=ultrafine opt=tight

phenol

C,-0.0055016859,0.0001155767,-0.0016686485\C,-0.00051398,0.0001279095,1.3
94106795\C,1.2095145754,0.0000811263,2.0898525462\C,2.4074548009,0.000
0224418,1.3838132103\C,2.4140393519,0.000010807,-0.0116564561\C,1.2020
268245,0.0000578238,-0.6973669445\O,-1.1450010245,0.0001811364,2.13113
00712\H,1.1912181917,0.0000918263,3.1751268238\H,3.3459898574,-0.00001
36911,1.9311637552\H,3.3528003713,-0.000034465,-0.556677595\H,1.189165
3936,0.0000491625,-1.7837385043\H,-0.9495421364,0.000150454,-0.5434454
33\H,-1.9085836131,0.0002340621,1.5449614737

phenoxyl

C,-0.0020125949,0.0000082187,-0.0036390634\C,0.000614378,-0.0000026
534,1.3727514195\C,1.2472141237,0.0000386821,2.110489246\C,2.475545826
4,0.0000724553,1.3427201641\C,2.4447742281,0.00008236,-0.0333288633\C,
1.2129337178,0.0000527598,-0.7146276807\O,1.2624349273,0.0000139155,3.
3648591548\H,3.4075459586,0.0000926266,1.8998081506\H,3.3707140281,0.0
001124825,-0.6017019911\H,1.1997549696,0.000059867,-1.8007157343\H,-0.
9414711401,-0.000018228,-0.5493772432\H,-0.9175938246,-0.0000385925,1.
9522900201

ortho-benzoquinone

C,-0.0049308641,0.,0.0575032316\C,-0.0523341172,0.,1.404
618097\C,1.1885277405,0.,2.1927825789\C,2.5315510166,0.,1.4071923254\C
,2.4527465351,0.,-0.0607077459\C,1.2553075824,0.,-0.6796625255\O,1.198
3063404,0.,3.4066313609\O,3.5848024652,0.,2.0106705315\H,3.3923234296,
0.,-0.605178243\H,1.20528614,0.,-1.765572832\H,-0.9269408342,0.,-0.518
3447668\H,-0.9874417382,0.,1.9567292701

ortho-benzosemiquinone

C,0.0005416729,0.,0.0368993582\C,-0.0153513294,0.,1.4
078051459\C,1.2221992051,0.,2.1401467962\C,2.4637600949,0.,1.364774940
5\C,2.4546275056,0.,-0.0242789965\C,1.2275290346,0.,-0.6777537641\O,1.
3256927292,0.,3.3898737148\O,3.5848752947,0.,2.0729859156\H,3.39157885
64,0.,-0.5717143823\H,1.2055694575,0.,-1.7635603767\H,-0.9325904298,0.
,-0.5187136016\H,-0.9387379174,0.,1.9782493453\H,3.2878409678,0.,3.008
2555223

para-benzoquinone

C,-0.0478169328,-0.0000117022,-0.0272090825\C,-0.0070376881,-0.0000
002743,1.4538033748\C,1.1587516076,-0.0000023696,2.1171657843\C,2.4527
98583,-0.000015665,1.3957093587\C,2.4120156674,-0.0000253543,-0.085301
6799\C,1.2462293872,-0.0000232596,-0.7486693888\O,3.5145743123,-0.0000
16945,1.9998887122\H,3.3748879176,-0.0000346181,-0.5886238147\H,1.1870
749249,-0.0000301051,-1.8335443216\O,-1.1095951978,-0.0000060991,-0.63
13898789\H,-0.9699136888,0.0000094553,1.9571150866\H,1.2178951925,0.00
00049412,3.2020428171

para-benzosemiquinone

C,0.0055838012,-0.0000067336,-0.011310343\C,-0.0027772945,0.
0000020764,1.3998333787\C,1.1819139477,-0.0000018841,2.088687539\C,2.4
579618998,-0.0000154008,1.4010294574\C,2.4148569088,-0.0000236159,-0.0
469574652\C,1.2210479036,-0.000019483,-0.7258847226\O,3.5443583172,-0.
0000182736,2.0246730604\H,3.3651167319,-0.0000332693,-0.571786403\H,1.
2037970041,-0.0000259629,-1.8145725305\O,-1.1926215705,-0.0000021446,-
0.6275355341\H,-0.961663784,0.0000119501,1.9092419256\H,1.2052639043,0
.0000048027,3.1740344243\H,-1.0782710272,-0.0000087631,-1.5847439458\

2,2’dimer DHN semiquinone “trans” conformer

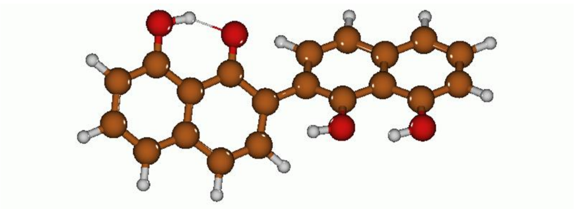

C,0.0172447687,0.360017309,0.0848164475\
C,0.0401425377,0.3412446348,1.4902418551\C,1.219303701,0.051162336,2.1
651213963\C,2.3949755428,-0.2236181768,1.4709938423\C,2.3984479774,-0.
2076744774,0.0657545023\C,1.2015729706,0.0845687218,-0.6406639653\H,-0
.8806712706,0.5593501803,2.0212655574\H,1.2207916321,0.0394619713,3.25
13000937\H,3.3122702078,-0.4511739602,2.0061517792\C,3.5741045961,-0.4
911529416,-0.6843700432\C,1.1715182617,0.1077425703,-2.0938981505\C,2.
4147074154,-0.1669938299,-2.8165097807\O,0.106519482,0.377639422,-2.70
72233479\C,2.4305340116,-0.1423101846,-4.2782670432\C,3.5682630974,-0.
4660929944,-2.0698047444\H,4.4841642213,-0.7566059419,-0.1534954052\H,
4.4754387942,-0.7497394994,-2.5990694164\C,3.4593210309,0.4919009187,-
4.9773245955\C,3.5326825266,0.4951667291,-6.3977737266\C,2.4971474938,
-0.1790395164,-7.1213487206\C,1.4361659681,-0.7903773353,-6.3984782694
\C,1.3931313768,-0.7581045788,-5.0350495291\O,4.4377395709,1.184233108

7,-4.3199903706\C,4.5777150075,1.1339783367,-7.1421159303\C,2.53559712
62,-0.2173792163,-8.5296717901\H,0.6501141275,-1.2903562869,-6.9569836
396\H,0.5727591979,-1.2208700387,-4.5002524736\C,3.5634701423,0.398187
8952,-9.2128352324\H,1.7432408829,-0.734160823,-9.0628507182\H,3.58959
10045,0.3675301257,-10.2984383662\C,4.579639309,1.073275152,-8.5249139
056\O,5.5942960617,1.8044621636,-6.5637943036\H,5.387916923,1.56645299
68,-9.0546564451\O,-1.1226563316,0.6399793209,-0.5322299104\H,-0.91561
44968,0.6065067418,-1.5141115966\H,5.4721172872,1.8087601786,-5.602023
7853\H,4.2174405018,1.2609218386,-3.3809446727

2,2’dimer DHN semiquinone “cis” conformer

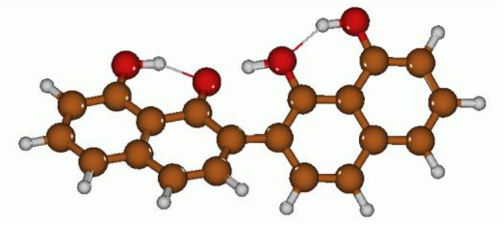

C,0.1285280795,-0.0069411119,-0.1550488315\C,0.03
61614652,-0.0813355449,1.2322209016\C,1.2239440716,-0.0432835376,2.031
7695439\C,2.4820159803,0.0625050235,1.3663359772\C,2.5389716803,0.1302
058828,-0.0344518054\C,1.3704691164,0.098693876,-0.778990807\C,3.66526
88961,0.0720628716,2.1587414733\C,3.6024781293,0.0305199408,3.51861772
56\C,2.3687118714,-0.0353016981,4.2409963213\C,1.1841502413,-0.1223811
315,3.4606579893\C,2.4264887633,0.0654181648,5.6945931124\C,1.48199806
18,-0.5415175131,6.6063352965\C,1.7322110002,-0.5135926159,8.026262100
4\C,2.8670596158,0.1661439782,8.5484496918\C,3.7382889049,0.8194266932
,7.629491492\C,3.522468326,0.7584297434,6.2787193333\C,0.8400558992,-1
.1524197936,8.9342991514\C,1.0954825511,-1.1103994969,10.3112675545\C,
2.2115828056,-0.4410175695,10.7968131834\C,3.0943839098,0.19831903,9.9
313137203\O,-0.2437717887,-1.8037024809,8.5236359373\O,0.4229026303,-1
.1463862299,6.1860875536\O,-0.0194255071,-0.2270742739,4.0136586616\O,
-1.1906374787,-0.1828115388,1.7549403919\H,0.3991192277,-1.6128126395,

10.9743902231\H,2.393544627,-0.4169194775,11.8674813125\H,3.961678569,
0.726137037,10.3168064335\H,4.5796026946,1.3856294825,8.0192292502\H,4
.1985688315,1.2971676122,5.6240419871\H,4.6285992602,0.0949950031,1.65
72330168\H,4.5287139043,0.0024690846,4.0818700908\H,3.5063461078,0.205
2442249,-0.5221122885\H,1.4171278543,0.1545668578,-1.8629164852\H,-0.7
929369139,-0.0342977848,-0.7270215811\H,-0.2650003996,-1.7201969747,7.
535414512\H,-1.0993905349,-0.2369433218,2.72856001\H,0.0713734892,-0.6
517761661,4.9745924071\

2,2'dimer 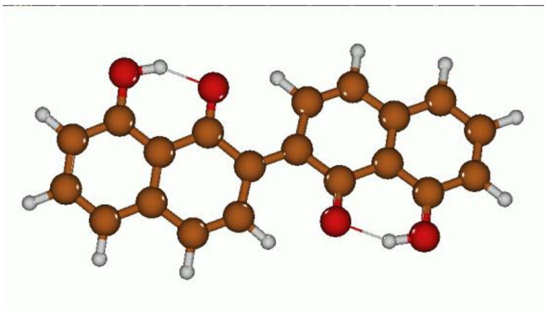 **DHN quinone**

C,-0.0632580013,-0.0788110074,0.1336739896\
C,-0.0096418663,-0.2001469841,1.5338209168\C,1.2133414146,-0.218072994
7,2.1804010081\C,2.4100052066,-0.1165915412,1.4625665432\C,2.378176321
7,-0.0021958931,0.0743504063\C,1.1400040851,0.0099007137,-0.6121686174
\H,-0.9457760798,-0.2694827555,2.0778059878\H,1.2426504398,-0.30735955
31,3.2627285124\H,3.3649256114,-0.1237178009,1.9796898114\C,3.58229681
72,0.1067111926,-0.7037834604\C,1.0825761139,0.1196403624,-2.062701218
4\C,2.3656171378,0.1839185432,-2.8378555224\O,-0.0330408871,0.19918127
79,-2.6210956229\C,2.3748400733,0.1852001347,-4.2417503136\C,3.5755652
204,0.2143669762,-2.0569116544\H,4.5330863885,0.0840028068,-0.17754947
12\H,4.5148474984,0.2905800927,-2.5826777955\C,3.6010782203,0.56800211
4,-5.016987698\C,3.5852525149,0.444449134,-6.4674599543\C,2.4276475983
,0.0047574437,-7.153861438\C,1.2600380865,-0.3088848674,-6.3756739918\

C,1.2290213398,-0.2048601378,-5.0226005554\O,4.6203802445,1.028554455,
-4.4587339333\C,4.7450727237,0.7767615515,-7.2133662155\C,2.4374469237
,-0.1142844126,-8.5420172197\H,0.3757544618,-0.6591146823,-6.901811050
8\H,0.3212481812,-0.457761201,-4.4967881765\C,3.595487139,0.2037786952
,-9.2599083923\H,1.5439000867,-0.4513160959,-9.0590509788\H,3.59894439
74,0.1093224853,-10.3421883935\C,4.7368279792,0.643670137,-8.613447362
1\O,5.8546944299,1.2136638026,-6.6318901921\H,5.6392713663,0.901927646
8,-9.1574817943\O,-1.2554500283,-0.0526928524,-0.44792231\H,-1.0768803
203,0.0617210024,-1.4234629405\H,5.6474966072,1.2597874538,-5.65639090
26

4,4'dimer DHN semiquinone

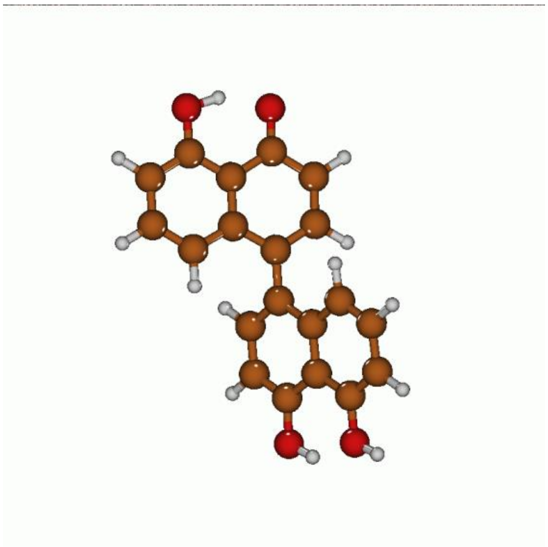

C,-0.0056336283,-0.5321485387,0.0854893259\C,-0. 0070643712, -0.1462226179,1.4044612019\ C,1.1992553579,0.0808381577,2.1275836784\C,2.4395258608,-0.08350225,1.4236545474\

C,2.4079033705,-0.511 6404324,0.0703467326\C,1.2183385428,-0.7266061921,-0.5799718001\C,3.6774071405,0.1373742561,2.1078131727\C,3.642828999,0.4576508073,3.4578840381\C,2.4417523904,0.6107508811,4.1536553129\C,1.22844535,0.4410811225,3.5128465412\H,2.434095783,0.8857394491,5.2030951325\O,0.1201070411,
0.6273192946,4.2533547654\O,-1.1844505094,0.0331914351,2.0861758068\C,
4.9721454942,0.1120204257,1.4049302746\H,4.5786928066,0.6350274686,3.9
802499756\H,3.3440681198,-0.6889163822,-0.4468869699\H,1.2147311939,-1
.0576044347,-1.6140629267\H,-0.9470902301,-0.6980348398,-0.4335590221\

H,-1.93704105,-0.1761323562,1.523315718\C,5.1780298386,0.9835836111,0.3126025071\C,6.3955923243,1.1095034671,-0.3134238146\C,7.5333106511,0.3383864623,0.116563257\C,7.3315450847,-0.5907328713,1.2162049722\C,6.0608395596,-0.7167368041,1.848856401\H,4.3468917157,1.6068053009,-0.007323529\H,6.5492359465,1.8086469892,-1.1294607324\O,8.6554594125,0.4625887193,-0.444308979\C,8.4133811983,-1.3990028321,1.6374429083\C,5.9018588003,-1.6826936871,2.8629612888\C,6.97998917,-2.4685810357,3.2607714292\H,4.9292406593,-1.8257892897,3.3198711952\H,6.8393865014,-3.2082953062,4.043965346\C,8.2293845113,-2.3332105401,2.6698790315\O,9.608289603,-1.2981753188,1.068856226\H,9.0750044915,-2.9420020278,2.9729336624\H,9.5150801193,-0.5899365438,0.3596366114\H,-0.6681407827,0.4745224153,3.709391041

4,4’dimer DHN quinone

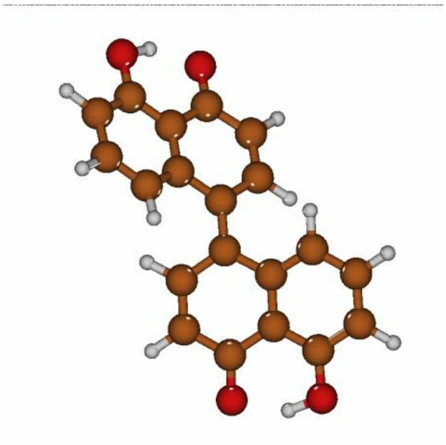

C,-0.1515413652,-0.1517017574,0.0781674176\C,-0.0441903655,0.243363511,1.4202672936\C,1.2171424294,0.216390556,2.0624948171\C,2.3755584606,-0.1735402303,1.3402965671\C,2.2250839082,-0.627413498,0.0233752242\C,0.9734697231,-0.5975871784,-0.5947704362\C,3.6689684749,-0.2076166244,2.0197337619\C,3.6708564502,-0.0563715811,3.4562865187\C,2.5627442811,0.3013393537,4.1501398391\C,1.2832764555,0.4951088136,3.4921366979\H,2.6040360608,0.5221505316,5.2120873801\O,0.2803525646,0.8692161722,4.1362810279\O,-1.1429262764,0.6168567904,2.0649960252\C,4.8955824494,-0.2272176431,1.3343712462\H,4.6255000623,-0.093466216,3.9694170953\H,3.0731155468,-1.0435672909,-0.5089837891\H,0.879180361,-

0.9457985937,-1.6194236504\H,-1.1291630482,-0.1287890694,-0.3919451813
\H,-0.8615303679,0.8269412627,3.0014574588\C,5.0242340123,0.5213851105
,0.1057109803\C,6.2288222524,0.8287073988,-0.4344972934\C,7.4694412367
,0.3671532151,0.1616456088\C,7.3685261316,-0.5056472139,1.3252461702\C
,6.1051375663,-0.8393287244,1.880266016\H,4.1289826378,0.9678926152,-0
.3128327422\H,6.315031295,1.486767321,-1.2934034777\O,8.5742904467,0.6
956360198,-0.3201737953\C,8.5476663151,-1.0992154329,1.8365727252\C,6.
0449932356,-1.8373129346,2.8617635201\C,7.2170657365,-2.4154424002,3.3
532311908\H,5.0845915745,-2.1957568463,3.2151676984\H,7.1497071444,-3.
1815407506,4.1204416377\C,8.4603944249,-2.0457243043,2.8687236326\O,9.
7444383502,-0.7968904,1.3479776622\H,9.3765728014,-2.4921658674,3.2409
892864\H,9.5892154283,-0.1371810877,0.6124652129

2,4’dimer DHN semiquinone

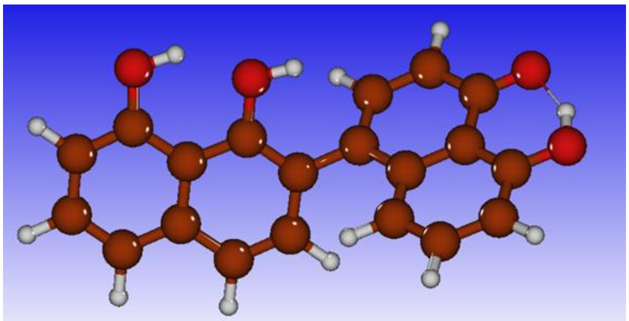

C,0,-0.0512141058,0.4631243471,0.10477096
68\C,0,-0.0317457255,0.1442544573,1.4732760325\C,0,1.2030825707,-0.065
8233094,2.1306214774\C,0,2.4221617436,0.0359472623,1.4003688804\C,0,2.
3704946875,0.3816033823,0.0356770995\C,0,1.1434563937,0.5852557939,-0.
5911107462\C,0,3.6692838799,-0.1766864001,2.0867818686\C,0,3.651942050
4,-0.4252561263,3.4780368734\C,0,2.4816039856,-0.5125541895,4.19914088
65\C,0,1.2017475718,-0.3490670902,3.5562512954\H,0,2.4800772203,-0.739
2043995,5.260632105\O,0,0.1301011708,-0.4449099888,4.2099973969\O,0,-1
.1825975512,0.0543233181,2.1254442114\C,0,4.9586153759,-0.1881613723,1
.3741808158\H,0,4.598820364,-0.6154934593,3.9793247537\H,0,3.291263449
5,0.5056231924,-0.5232268331\H,0,1.121243013,0.8509566864,-1.644125109
7\H,0,-1.0116784913,0.6205602839,-0.374994309\H,0,-0.945555342,-0.1665

559237,3.0769854513\C,0,5.9975324783,0.6453259996,1.7723221877\C,0,7.2
724885043,0.633849644,1.1389399387\C,0,7.4720487068,-0.2829730349,0.05
6657057\C,0,6.3992071359,-1.1247563498,-0.3439305982\C,0,5.1865381298,
-1.0722898782,0.284050676\O,0,5.8250225795,1.5482055164,2.7840071684\C
,0,8.3599445241,1.4853967475,1.5194046451\C,0,8.7214530889,-0.33728601
75,-0.5968234398\H,0,6.5638331524,-1.8240031749,-1.1586638855\H,0,4.38
13800907,-1.7350372262,-0.0189574905\C,0,9.5689715751,1.401739326,0.85
23137129\O,0,8.2788800166,2.3868557668,2.5188588\H,0,10.370725892,2.06
09949237,1.1682269991\C,0,9.7469446174,0.4925601426,-0.1994326429\H,0,
8.8570733577,-1.039643932,-1.4138161842\H,0,10.7079433973,0.449536367,
-0.704076824\H,0,7.3881908325,2.370518933,2.9008162021\H,0,4.905560942
2,1.541589763,3.0849118371\

2,4'dimer DHN quinone

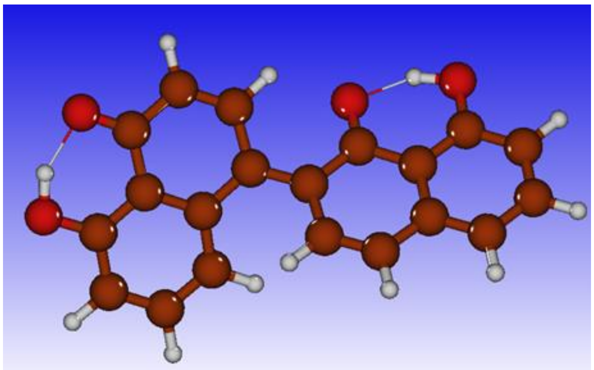

C,0,-0.0607367341,-0.2033681925,0.1565649214
\C,0,0.0163302721,-0.0841009312,1.5475917091\C,0,1.2603272197,0.016946
7217,2.1683084721\C,0,2.4460066405,-0.0081396358,1.3910344867\C,0,2.34
62221208,-0.1219832195,-0.0206023681\C,0,1.0819543483,-0.2223036679,-0
.624776398\C,0,1.3826680532,0.1273262227,3.5983471576\C,0,2.5864283386
,0.2438524796,4.2113755211\C,0,3.8362193141,0.2146291634,3.494420013\C
,0,3.7586632565,0.0822567197,2.010321959\C,0,5.0619351351,0.2547885983
,4.1708792584\C,0,5.2121892205,0.8460589864,5.5057580146\C,0,6.3057266
802,0.4545525811,6.3213672678\C,0,7.3464399982,-0.4246757087,5.8013983
548\C,0,7.2706609072,-0.7824863152,4.3945665503\C,0,6.21648387,-0.4292
76069,3.6228518786\C,0,4.370739839,1.8664077103,5.962883589\C,0,4.5546

976821,2.4211194957,7.2324001162\C,0,5.5700317422,1.9886250836,8.06678
27269\C,0,6.4662112763,1.0081867022,7.6128070855\O,0,7.4644282607,0.64
0951337,8.4080844368\O,0,8.2912775154,-0.8192505054,6.5145265182\O,0,4
.7829606024,0.1176528284,1.2922075807\O,0,3.4201082385,-0.1372146261,-
0.7996635033\H,0,8.0830002186,-1.3858874887,4.0013584555\H,0,6.1718561
751,-0.7451144678,2.5898115841\H,0,3.596183093,2.2628077892,5.31676649
47\H,0,3.8905221502,3.213466654,7.5657320965\H,0,5.7181611398,2.408196
0887,9.0564016216\H,0,8.0029532641,-0.0256067853,7.894743694\H,0,0.476
3020444,0.076540824,4.1956178644\H,0,2.6235317832,0.2621263275,5.29446
9127\H,0,1.0348616306,-0.3067665644,-1.7053514017\H,0,-0.8875971371,-0
.0678498776,2.1492584383\H,0,-1.0333709357,-0.2789903968,-0.3215728595
\H,0,4.2024813405,-0.0349684715,-0.1873549235\

2,2' dimer DHI semiquinone

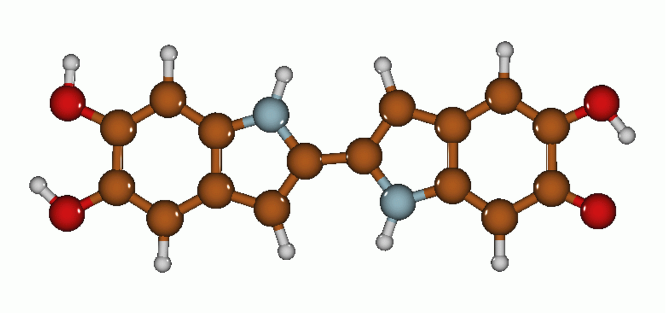

C,0.0012146135,0.0288275453,0.0001871413\C,0.002159
8293,0.0651100102,1.3784839208\C,1.2336086956,0.0424559254,2.058758981
3\C,2.4391913899,-0.0288514283,1.3157793061\C,2.4496384213,-0.06453826
42,-0.0815341229\C,1.222613094,-0.0338870139,-0.7198710074\C,1.5998875
682,0.07275533,3.4331578761\C,2.9827160794,0.0173973256,3.498101139\N,
3.4785195784,-0.0584486519,2.2094123969\C,3.8550829211,0.0116371407,4.
6324728016\C,5.2141180015,-0.2946633131,4.7159979041\C,5.6086247144,-0
.1493974687,6.0587115311\C,4.4386184008,0.2611693764,6.7965911763\N,3.
400352132,0.3280092963,5.8920928642\C,6.8431011836,-0.3304459513,6.725

1674535\C,6.8782211115,-0.085871291,8.0718477683\C,5.6878681744,0.3490
144466,8.8410594481\C,4.4474736583,0.5138632746,8.1457996998\O,5.85515
99957,0.5399747526,10.0674909406\O,7.9826401482,-0.2157619653,8.808559
5934\O,1.0655193448,-0.0623502061,-2.0775493092\O,-1.1727967463,0.0531
742355,-0.681309493\H,3.5716610478,0.8224642943,8.7070783542\H,7.73510
45358,-0.6471759336,6.1947100988\H,5.83781959,-0.630535359,3.898628726
1\H,2.4883616578,0.7031054272,6.0909463383\H,0.9237831754,0.0868727581
,4.2773057724\H,4.4537791842,0.0057878905,1.9717227755\H,3.3745535877,
-0.1185064088,-0.650583508\H,-0.9440266099,0.1130088739,1.9074400015\H
,-0.9787866912,0.0193276706,-1.6269078217\H,7.6915526388,0.0256047578,
9.7149362036\H,1.9183566832,-0.1113925122,-2.5198990261\

2,2' dimer DHI quinone

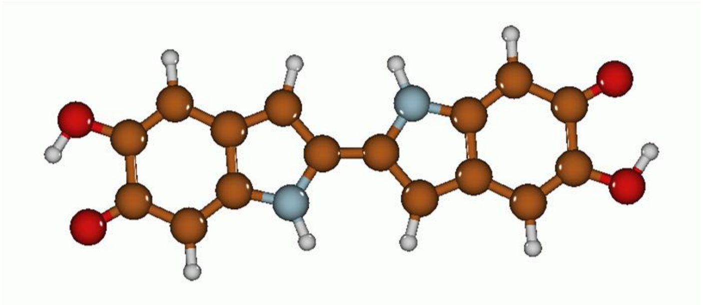

C,0.1149166884,0.,-0.3010264282\C,-0.2119317666,0.,1.0259
966763\C,0.8663226236,0.,1.9717055315\C,2.2750604842,0.,1.4560873735\C
,2.5777908,0.,0.1314107997\C,1.4904575871,0.,-0.7902853122\H,-1.231320
313,0.,1.3963715145\O,0.7407103132,0.,3.2060614008\O,3.1869481219,0.,2
.4234979934\H,3.607378152,0.,-0.2101987627\C,1.4329249285,0.,-2.165512
8467\C,0.0618414144,0.,-2.5636719901\H,2.278907253,0.,-2.8400498096\C,
-0.4828799407,0.,-3.8333909283\N,-0.697456046,0.,-1.4037229732\H,-1.70
24155942,0.,-1.371076872\C,-1.8539626097,0.,-4.2315502152\N,0.27642075
29,0.,-4.9933383894\H,-2.6999427471,0.,-3.5570116051\C,-0.5359487841,0
.,-6.0960350285\C,-1.9114924391,0.,-5.6067803567\C,-0.2091003355,0.,-7
.4230597874\C,-2.9988244286,0.,-6.528476719\H,1.2813802604,0.,-5.02597

50269\C,-1.2873538866,0.,-8.3687670434\H,0.8102897381,0.,-7.7934274475
\O,-1.1617415332,0.,-9.6031242218\C,-2.6960896141,0.,-7.8531526498\H,-
4.0284129505,0.,-6.1868733211\O,-3.6079754285,0.,-8.8205641577\H,-3.07
85609273,0.,-9.6490755306\H,2.6575337317,0.,3.2520102652

2,4’ dimer DHI semiquinone

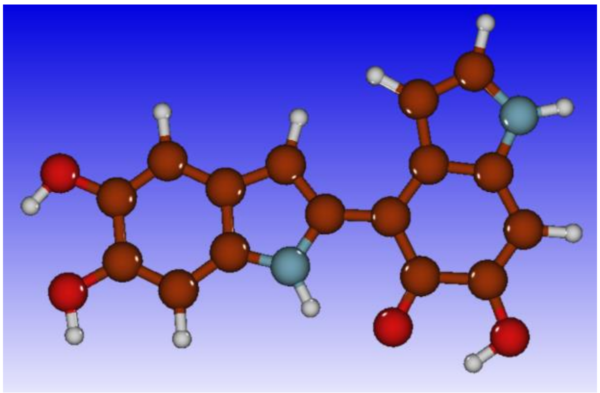

C,-4.3951273304,0.4411847741,-0.0086200929\C,-3.2
257395365,1.1412433851,0.1787697484\C,-1.99737758,0.4543745048,0.07780
97978\C,-4.3635704226,-0.9515956194,-0.3001575647\C,-3.1787043975,-1.6
565847828,-0.4061655112\C,-1.9938408218,-0.9393124041,-0.2146080191\N,
-0.6988201217,-1.3546885816,-0.2582453905\C,-0.6468292169,0.8452706605
,0.2010873331\C,0.1480441973,-0.2915936928,-0.0107658305\O,-5.59961340
22,-1.510292079,-0.4611316084\O,-5.5953491658,1.0717650886,0.082060282
1\H,-0.3161369697,-2.2630667835,-0.5050711734\H,-6.2933698415,0.424115
4026,-0.0792174632\H,-3.1761376838,-2.7204208953,-0.6296477891\H,-3.27
1811678,2.202633547,0.3997296851\H,-0.2936183941,1.846785242,0.3841444
476\C,1.5703152081,-0.4575815548,-0.0269532986\C,2.4930487156,0.554385
9786,0.343673048\C,2.1321896462,-1.7287973027,-0.4640971357\C,3.587510
531,-1.8893507842,-0.5726308858\C,3.8842632983,0.3138626818,0.21397802
53\C,4.4581613668,-0.8913793478,-0.2503939603\C,2.3667629918,1.8813024
956,0.8838912157\N,4.5394441335,1.431985965,0.6336597938\C,3.627583841
7,2.3812902818,1.0452843962\O,1.4525004541,-2.7462922413,-0.7836445133
\O,3.9863938351,-3.0913137525,-1.0073913552\H,5.53979446,1.5368290831,
0.6588515598\H,3.1528785679,-3.5847996905,-1.1495167441\H,5.5309100252

,-1.0311572525,-0.3365889158\H,1.4647397665,2.4047622305,1.1558532985\
H,3.9562834781,3.3365840708,1.4277345382\H,-5.5247279548,-2.449348627,
-0.6572299176

2,4’ dimer DHI quinone

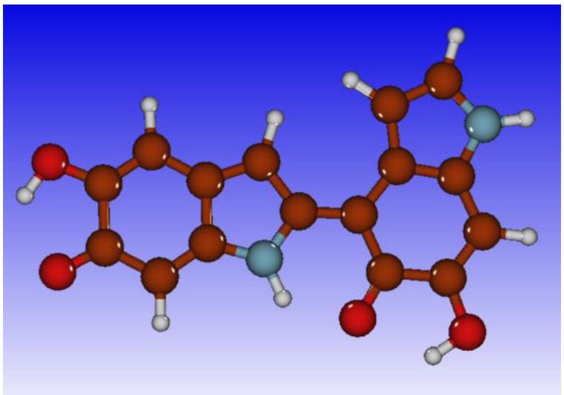

C,-4.3543142838,0.5253409768,0.02355129\C,-3.1994042982,
1.1999051494,0.2429209723\C,-1.9754708565,0.472737043,0.1085037144\C,-
4.3842706155,-0.9306289825,-0.3516807816\C,-3.1381579517,-1.6442334814
,-0.4852380702\C,-1.9917915828,-0.9476639976,-0.2581520344\N,-0.689210
1974,-1.3594738538,-0.3146748133\C,-0.6648247168,0.8342842856,0.250611
9403\C,0.1610916345,-0.3241823917,-0.0145448237\O,-5.5032014497,-1.425
4369564,-0.5258597945\O,-5.5635894059,1.0724054389,0.1151974527\H,-0.3
032467637,-2.2695501005,-0.5643837794\H,-6.1837002494,0.3428524562,-0.
0981011975\H,-3.1641214399,-2.6926135747,-0.760656035\H,-3.202649564,2
.2507258474,0.5119691401\H,-0.2981495445,1.8182020461,0.4926750678\C,1
.5481608917,-0.4617751511,-0.0143212266\C,2.4794375186,0.5811489024,0.
344691689\C,2.1108646408,-1.7519468012,-0.4394787157\C,3.5626954044,-1
.90425236,-0.5475618414\C,3.8523929518,0.3304039578,0.2128338017\C,4.4
16851312,-0.8938185071,-0.2369693783\C,2.3632712741,1.9096639397,0.862
6489777\N,4.5275110412,1.447845398,0.6145086477\C,3.6345356225,2.40786
12554,1.0121300832\O,1.4282676757,-2.7616311509,-0.7456262288\O,3.9773
035065,-3.1107021507,-0.9686942492\H,5.5292674775,1.5425500068,0.62961
04684\H,3.1563066061,-3.6204603229,-1.1095484405\H,5.4905619498,-1.032
8048239,-0.3229368687\H,1.4711349424,2.4540412099,1.1282819995\H,3.969

0784701,3.3683366928,1.3752730341

4,4’ dimer DHI semiquinone

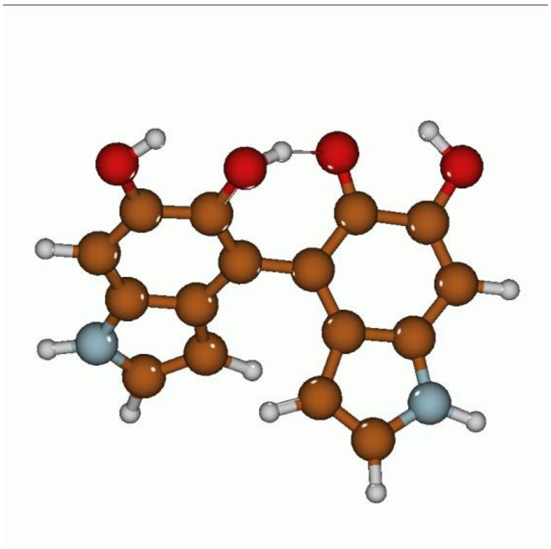

C,-0.0868919366,0.0989695164,0.0073011824\C,-0.18157
80381,-0.1267449778,1.3930216134\C,1.0089486541,-0.2276823465,2.058535
9265\C,2.3090763483,-0.1206404705,1.3758688674\C,2.3725467272,0.021437
6827,-0.0624598388\C,1.1434025432,0.1715796486,-0.7200569756\H,-1.1289
395207,-0.1893655752,1.9177643324\O,1.0823532722,-0.4039028744,3.37852
74718\O,3.318548303,-0.1682819737,2.1407429294\C,3.649976643,-0.013805
9484,-0.7846615239\C,0.7887494898,0.4717793224,-2.0846244379\C,-0.5676
641084,0.5533006497,-2.1410448779\H,1.4709750397,0.6099502488,-2.90930
73052\H,-1.2214190395,0.7659648383,-2.9746233732\N,-1.093923209,0.3172
251157,-0.8776984369\H,-2.0718422162,0.3462064841,-0.6419461603\C,3.78
77750527,-0.7703200806,-1.9873995845\C,5.0021078091,-0.7143183253,-2.7
109062673\C,6.0989969345,0.0493832977,-2.3066644546\C,5.9719510648,0.7
400114112,-1.1168357848\C,4.7803722894,0.690147984,-0.3508978701\C,2.9
51728117,-1.7130042056,-2.6693792221\H,7.030279687,0.0910233235,-2.862
3973918\O,7.0016292081,1.4823058567,-0.6476922361\O,4.8373056407,1.431
0346218,0.7884050387\C,3.652950932,-2.1672849026,-3.7559146022\H,1.953
9521547,-2.0212575092,-2.394662907\H,3.3692163745,-2.882586117,-4.5148
076579\N,4.8834714344,-1.5581710509,-3.788720013\H,5.6085501787,-1.744
914234,-4.4596627522\H,6.7115239219,1.8365149561,0.2080390082\H,2.0420

226687,-0.4160355054,3.5741212199\H,4.3740024333,0.9134141728,1.492217
237

4,4' dimer DHI quinone

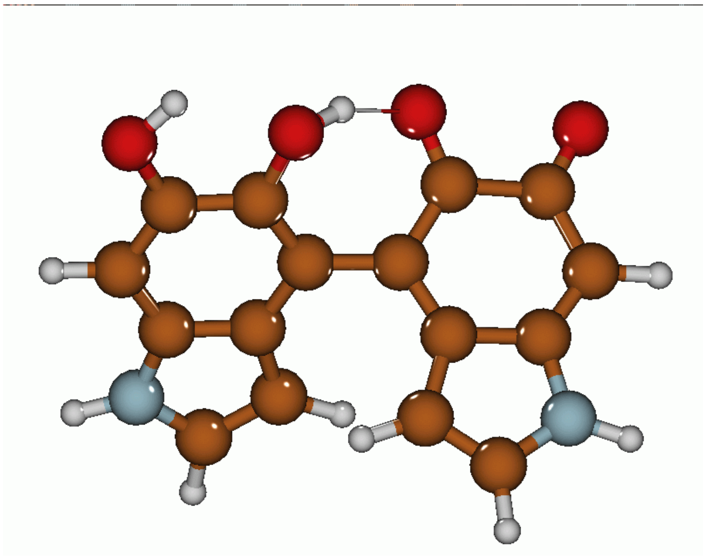

C,-0.1135993923,0.0816265631,0.0388772174\C,-0.256767052,
-0.2546261959,1.3458551928\C,0.9543038152,-0.47943421,2.1029232639\C,2
.3268103153,-0.1932337279,1.4025797763\C,2.3879084821,-0.0035500081,-0
.0528107604\C,1.1775729434,0.1971800677,-0.6742733403\H,-1.2149577118,
-0.3936211934,1.8353929844\O,0.9742499635,-0.8573073318,3.260177652\O,
3.3046277803,-0.1088725209,2.1360219961\C,3.6630600637,-0.0125733663,-
0.7914746392\C,0.8506536171,0.566895894,-2.0285812282\C,-0.5008836816,
0.656168854,-2.106918068\H,1.5538750843,0.7432222592,-2.8275188999\H,-
1.1201376727,0.9222199316,-2.9533735242\N,-1.0879826572,0.3523662869,-
0.8900632776\H,-2.072915568,0.3926598433,-0.6916253447\C,3.7953172497,
-0.7928060837,-1.9756019892\C,5.0009898281,-0.7391328201,-2.7145290634
\C,6.0927382821,0.0445472051,-2.3366828094\C,5.9660335794,0.7660903777
,-1.164955936\C,4.7788059942,0.7252440889,-0.3910605182\C,2.9509250578
,-1.7421241022,-2.6389692769\H,7.0190400602,0.0836044134,-2.900703471\
O,6.9896358641,1.5343105011,-0.7260794084\O,4.8263132042,1.5113911185,
0.7221713855\C,3.64158314,-2.2072640356,-3.7272695626\H,1.9569137206,-
2.0505181261,-2.3498841107\H,3.3517574722,-2.9321871552,-4.4746593411\
N,4.8717003916,-1.5977133971,-3.7795787049\H,5.5877061262,-1.788676432

6,-4.4590905988\H,4.3849333779,1.0168009967,1.4468672431\H,6.704667448
8,1.911245339,0.1213516487
